# Recombination and Population Structure in *Salmonella enterica*

**DOI:** 10.1371/journal.pgen.1002191

**Published:** 2011-07-28

**Authors:** Xavier Didelot, Rory Bowden, Teresa Street, Tanya Golubchik, Chris Spencer, Gil McVean, Vartul Sangal, Muna F. Anjum, Mark Achtman, Daniel Falush, Peter Donnelly

**Affiliations:** 1 Department of Statistics, Oxford University, Oxford, United Kingdom; 2 Wellcome Trust Centre for Human Genetics, Oxford University, Oxford, United Kingdom; 3 Strathclyde Institute of Pharmacy and Biomedical Sciences, University of Strathclyde, Glasgow, United Kingdom; 4 Veterinary Laboratories Agency, Addlestone, United Kingdom; 5 Environmental Research Institute and Department of Microbiology, University College Cork, Cork, Ireland; 6 Max Planck Institute for Evolutionary Anthropology, Leipzig, Germany; Universidad de Sevilla, Spain

## Abstract

*Salmonella enterica* is a bacterial pathogen that causes enteric fever and gastroenteritis in humans and animals. Although its population structure was long described as clonal, based on high linkage disequilibrium between loci typed by enzyme electrophoresis, recent examination of gene sequences has revealed that recombination plays an important evolutionary role. We sequenced around 10% of the core genome of 114 isolates of *enterica* using a resequencing microarray. Application of two different analysis methods (Structure and ClonalFrame) to our genomic data allowed us to define five clear lineages within *S. enterica* subspecies *enterica*, one of which is five times older than the other four and two thirds of the age of the whole subspecies. We show that some of these lineages display more evidence of recombination than others. We also demonstrate that some level of sexual isolation exists between the lineages, so that recombination has occurred predominantly between members of the same lineage. This pattern of recombination is compatible with expectations from the previously described ecological structuring of the *enterica* population as well as mechanistic barriers to recombination observed in laboratory experiments. In spite of their relatively low level of genetic differentiation, these lineages might therefore represent incipient species.

## Author Summary

*Salmonella enterica* is a species of bacteria that causes severe diseases in humans and animals. We sequenced about a tenth of the genome from a broadly sampled collection of *S. enterica*. By comparing these genetic sequences, we were able to partially reconstruct the ancestry of this sample. We identified five lineages within *S. enterica*, one of which is almost as old as the common ancestor of our sample. We also found evidence for frequent homologous recombination in the ancestry of *S. enterica*, where fragments of genes from one individual bacterium are acquired by a distinct individual. These recombination events make the ancestry harder to reconstruct in its entirety, but also contain interesting information. We found in particular that recombination had happened more often between strains belonging to the same lineage than across lineage boundaries. This observation is compatible with the lineages of *S. enterica* becoming progressively isolated from each other, which could lead to their gradual splintering into new species.

## Introduction

*Salmonella enterica* subspecies *enterica* (subsequently referred to simply as *enterica*) is a major cause of enteric fever in humans and gastroenteritis in humans and animals. Its diversity has traditionally been described on the basis of serological differences following the Kauffmann-White classification [1], [2]. Certain serovars are linked to particular diseases and hosts. For example, enteric fever is mostly caused by members of serovar Typhi and Paratyphi A, both of which only infect humans [3]. Gastroenteritis on the other hand is most often caused by Enteritidis in humans and Typhimurium in animals [4], although both serovars can infect a wide range of hosts [3]. However, the usefulness of the serological classification of *S. enterica* is undermined by the fact that unrelated strains sometimes belong to the same serovar [5], [6].

In an attempt to shed some new light on the population structure of *enterica*, a multi-locus sequence typing scheme (MLST; [7], [8]) was developed which relies on the sequencing of 400-500 bp fragments from seven housekeeping genes. This typing technique was originally applied to strains from serovar Typhi [9], and later to the whole of *enterica* [10], [11]. Phylogenies reconstructed from MLST data are highly star-shaped [12] and therefore carry little information about relationships between isolates. This can be traced back to substantial incongruencies between gene trees [13], [12], [14], which are often caused by high levels of homologous recombination [15]. This is in contrast for example with the closely related species *Escherichia coli* which has a well defined population structure made of several clearly defined clades [16].

The first genomes of *enterica* to be fully sequenced were those of Typhimurium LT2 [17] and Typhi CT18 [18], followed by those of Typhi Ty2 [19], Paratyphi A [20] and Choleraesuis [21]. A comparison of the genomes of Typhi and Paratyphi A revealed that they had exchanged about a quarter of their genes during the course of their adaptation to a human-specific and highly virulent lifestyle [22]. This high level of recombination is, however, exceptional between two distantly related lineages of *enterica* [22], and selection is likely to have favoured recombinants between these two types which combined adaptations to their new host [22]. The pattern of recombination of these strains, with a burst of recombination being followed by completely clonal evolution [23], [24], appeared to be atypical of gene flow in the species as a whole, but only limited data from a small number of lineages has been analyzed [22]. The number of *enterica* genomes currently available is insufficient (only eleven whole published genomes available at the time of writing in the Genomes OnLine Database; [25]), and their distribution is too focused on highly virulent types to allow an exploration of the population genetics of *enterica*. Furthermore statistical methodology to analyze such whole-genome data efficiently is currently lacking [26], [15].

Reconstructing the clonal relationships between lineages that have evolved under the influence of recombination requires data from a large number of loci [27]. We therefore designed an Affymetrix CustomSeq Resequencing Array to sequence approximately 300Kbp from the core genome of *enterica* isolates, which represents two orders of magnitude more data per isolate than is provided by MLST. Resequencing arrays are a highly parallel DNA sequencing technology with quick application and low cost, and are based on the principle of sequencing by hybridization [28]. They have been previously applied to a wide diversity of bacterial samples, including monomorphic clones such as *Bacillus anthracis* [29] or *Mycobacterium tuberculosis* [30], relatively clonal species such as *Bacillus cereus* [31] or *Staphylococcus aureus* [32], and species with high rates of recombination such as *Neisseria meningitidis* [33] or *Francisella tularensis* [34].

We applied our resequencing array to a global collection of 114 isolates from multiple major lineages of *enterica*, with the exception of Typhi. Typhi was excluded because extensive studies using a wide range of molecular techniques [23], [35], [24], [36], [37] have revealed that its population biology differs from that of other lineages of *enterica*. We therefore excluded Typhi from the present study in order to focus on the remainder of *enterica*, which has been studied much less thoroughly. The main aims of this study were to provide an improved description of the population structure of *enterica* and to clarify the role played by recombination during its evolution. To this end, we analyzed our genetic data using the linkage model of Structure [38], [39] and ClonalFrame [40] with *a posteriori* attribution of the origin of recombination events [41].

## Results

### Novel nucleotide sequences

**Figure 1:**
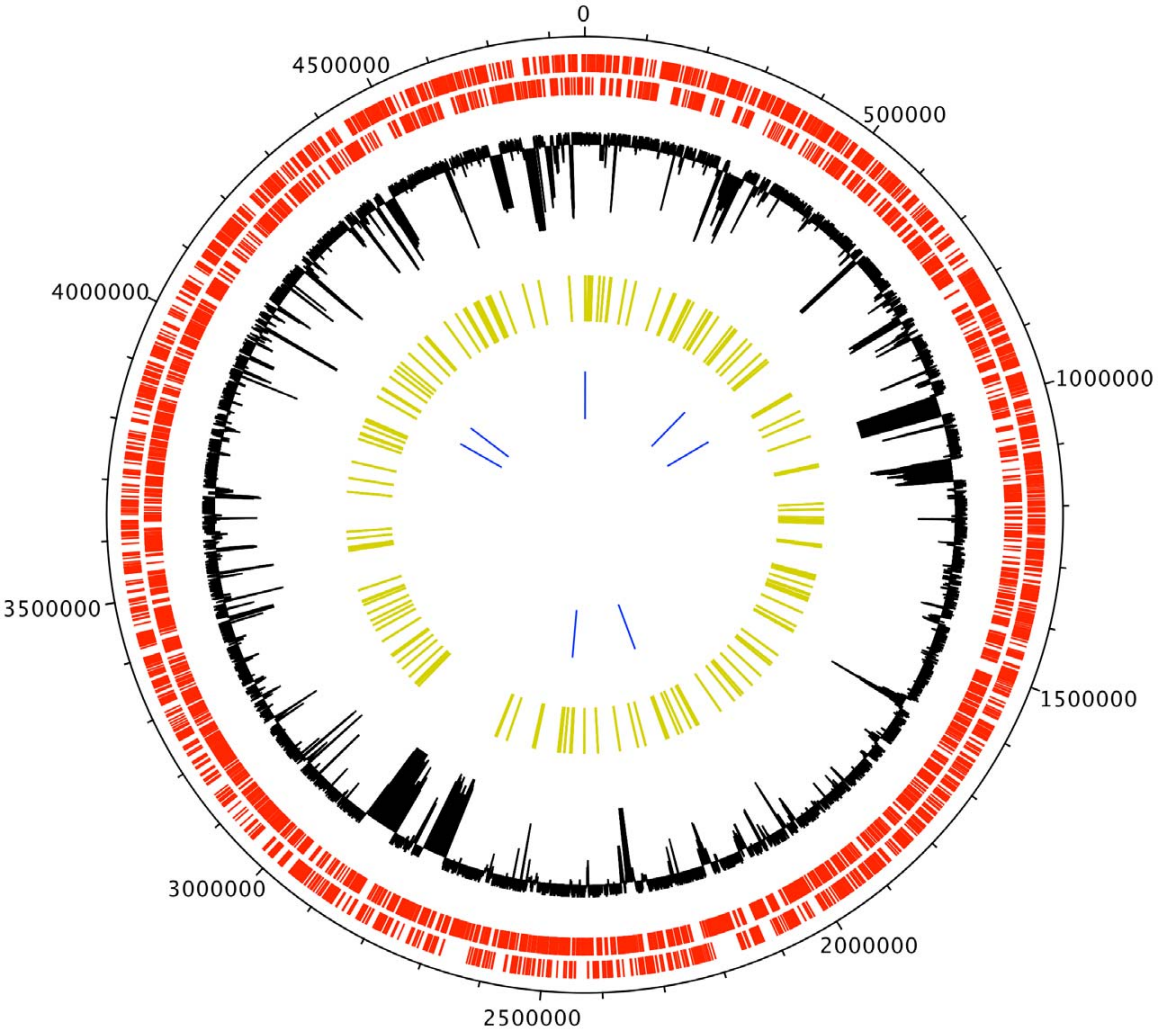
The circle represents the Typhimurium LT2 genome [**17**]. The two circles in red represent the coding regions, with the forward strand on the outside and the reverse strand on the inside. The black circle indicates the proportion of 10 other genomes that aligned to each specific region of LT2, with proximity to the center indicating less genomes aligning. The yellow bars represent coverage of our sequencing scheme, and the blue bars coverage of the MLST scheme. This Figure was drawn using DNAPlotter [82].

For each of the 114 isolates under study (Table S1) we resequenced 146 regions of length 2000-2500bp each from the core-genome of *enterica* (Table S2). These 295,137 bp per isolate represent approximately 10% of the core genome of *enterica* [42]. Figure 1 illustrates the extent of our resequencing scheme on the genome of Typhimurium LT2 [17]. On average, 85% of nucleotides were called, with variation across isolates ranging from 75% to 95%. A total of 18,068 of the resequenced sites (6%) were found to be polymorphic in this sample. Regions overlapping the seven MLST loci were included in our resequencing scheme, and by comparing our results with preexisting MLST sequences we estimated the error rate of our method to be lower than one error per 10,000 calls. Only one isolate had more than one error in its MLST gene fragments: isolate 54 (SARB32; ST82) had two errors, one in gene *hisD* and the other in gene *purE*. An equivalent error rate was found when comparing the sequence of LT2 reported in [17] with our resequenced sequence of LT2. The density of errors was therefore sufficiently low enough that errors would be misinterpreted as mutations, and would not affect our results below which are essentially focused on the recombination process.

### Population structure of *Salmonella enterica*

**Figure 2:**
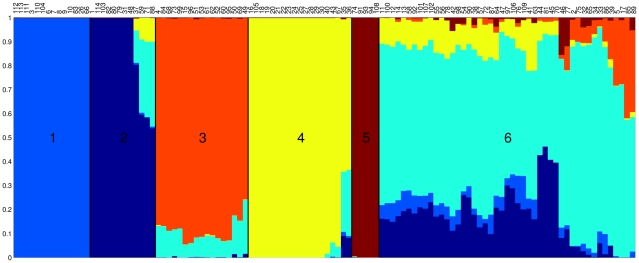
Result of applying the linkage model of Structure to our data assuming *K = 6* populations. Each vertical line represents one of the 114 isolates, ordered on the X axis by the proportion of ancestry from the major ancestral source. The colouring of each vertical line is proportional to the ancestry of each isolate from each of the 6 populations using the following colours: light blue, dark blue, orange, yellow, dark red and cyan representing ancestral populations 1 to 6, respectively.

We applied the linkage model of Structure [38], [39] to our data and identified 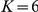 ancestral populations in our sample (Figure S1). The proportion of ancestry from each of these sources is shown for each isolate in Figure 2. The 114 isolates fell into six distinct groups based on the major ancestral source of genetic diversity of each isolate. (Figure 2). Group 1 (light blue) consisted of 14 strains of Choleraesuis, Paratyphi C and Typhisuis, Group 2 (dark blue) comprised 12 strains of Typhimurium and Saint-Paul, Group 3 (orange) contained 17 strains of Montevideo, Javiana, Decatur and others, Group 4 (yellow) consisted of 19 strains of Enteritidis, Gallinarum and Dublin and Group 5 (red) comprised 5 strains of Paratyphi A and Sendai. Finally, Group 6 (cyan) contained the remaining 47 strains from diverse serovars. These groups showed relatively little admixture between ancestral sources (Figure 2), with the exception of Group 6, which seemed to have acted frequently both as a donor and as a recipient of recombinational exchanges (Figure 2).

**Figure 3:**
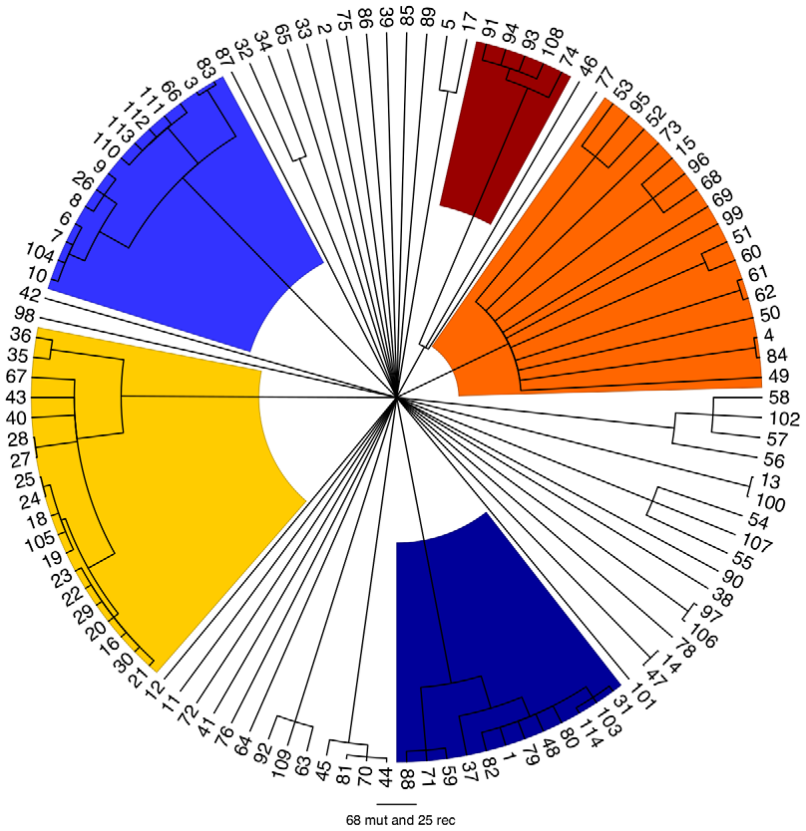
Clonal genealogy inferred by ClonalFrame from our data. The first five populations identified in Figure 2 by Structure corresponded to clades of the ClonalFrame clonal genealogy and have therefore been coloured with the same colours as in Figure 2. This figure was drawn using FigTree [83].

ClonalFrame is a method designed to reconstruct the clonal relationships between isolates in a sample, while accounting for the effect of non-vertical genetic transfer which would otherwise confuse such a reconstruction [40]. Figure 3 shows the clonal genealogy inferred from our data by ClonalFrame. The first five groups identified by Structure (Figure 2) corresponded to clades on Figure 3 and are represented with corresponding colors. Based on the combined evidence from the Structure and ClonalFrame analyses, these five groups can confidently be called lineages of *enterica*. On the other hand, the sixth group found by Structure encompassed the remaining isolates in Figure 3, which did not constitute a clade in Figure 3 and therefore did not represent a true lineage. Instead, seven small groups of two to four isolates formed small clades at this level of analysis according to ClonalFrame, but these were not detected by Structure. The content of the five identified lineages of *enterica* is summarized in Table 1.

**Table 1:** Content of the lineages and results of the ClonalFrame analysis.

	Lineage 1	Lineage 2	Lineage 3	Lineage 4	Lineage 5
Color in the Figures	Light Blue	Dark Blue	Orange	Yellow	Red
Isolates	14	12	17	19	5
Serovars	Choleraesuis	Typhimurium	Montevideo	Enteritidis	Paratyphi A
	Paratyphi C	Saint-Paul	Javiana	Gallinarum	Sendai
	Typhisuis		Decatur	Dublin	
			…		
MLST Sequence Types (STs)	66,68,90,114	19,27,36,50	4,20,23,24,48	10,11,73,78,92	85
	133,139,145	98,99	65,70,79,80,81		
	146,147		93,94,96,138		
eBURST MLST groups	6,20	1,14,138	40,12,41,17,42	53,4	11
			43,133,33,39		
			prov50,prov111		
Age relative to TMRCA of *S. enterica*	0.15	0.2	0.66	0.23	0.08
Mutation events	624	467	1879	736	192
Recombination events	48	178	1140	144	14
Substitutions introduced by rec	122	1013	5551	604	28
Relative frequency of rec and mut	0.08	0.38	0.61	0.20	0.07
Relative effect of rec and mut	0.20	2.17	2.95	0.82	0.15

Using Structure and ClonalFrame on MLST data only revealed parts of this population structure, and hardly revealed any relationships within lineages in comparison with the resequencing array data (Figures S3 and S4). Yet the deep phylogeny of *enterica* remained largely unresolved when using our resequencing data, and in particular the relationships of the five lineages above with one another and with the rest of the isolates remained unclear (Figure 3). We estimated the age of the five lineages relative to the time of the most common ancestor of the whole of *enterica* (Table 1). The common ancestor of lineage 5 was the most recent, followed by that of lineage 1. Lineage 3 was found to be particularly ancient, with an estimated age of two thirds of the age of *enterica*.

### Uneven role of recombination in *enterica*

Widespread recombination has previously been suggested to explain the lack of deep structure in *enterica* [12], [14] and we wanted to assess the role played by recombination in the evolution of *enterica*. Measuring the frequency of recombination is often done relative to that of mutation [43] by forming the ratio 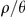 of rates at which recombination and mutation occurred in the ancestry of a sample. ClonalFrame estimated that recombination happened less frequently than mutation with 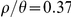 (95% credibility interval 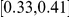). Recombination can however change several nucleotides in a single event. Another measure of recombination is therefore the ratio 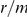 of rates at which substitutions are introduced by recombination and mutation [44]. ClonalFrame estimated that recombination and mutation had approximately the same effect in introducing polymorphism with 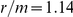 (95%CI [1.06, 1.23]). Recombination was found to affect segments of length 1826 bp on average (95%CI [1670, 1980]) which is comparable to the lengths of recombination tracts estimated when comparing four genomes of Typhimurium [40] as well as the lengths of the regions that were exchanged by Typhi and Paratyphi A [22].

We further studied recombination by looking at its specific role and patterns within each of the five lineages of *enterica*. The role played by recombination seems to be uneven across these five lineages according to the Structure results in Figure 2. The isolates in recently diversified populations 1 and 5 showed no admixture (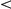
1% of material from other populations) whereas the isolates in population 4, 3 and 2 had acquired 4%, 11% and 12% respectively of their genetic material from a different population (Figure 2). To confirm this observation, we extracted from ClonalFrame output the numbers of mutation events, recombination events, and substitutions introduced by recombination for each of the five lineages (Table 1). Recombination was found to have played a much more important role relative to mutation in lineages 2 and 3 (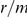 = 2.17 and 2.95 respectively) than in lineages 1 and 5 (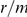 = 0.20 and 0.15 respectively), and a somewhat intermediate role in lineage 4 (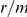 = 0.82). These results are in good qualitative agreement with those of Structure (Figure 2). Since lineages 1 and 5 are the most recently evolved from a common ancestor, these results point to a possible reduction in the role played by recombination in these two lineages, and maybe even throughout *enterica*.

### Patterns of genetic flux in *enterica*

ClonalFrame estimated that within the regions imported by recombination, an average of 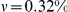 of the nucleotides were substituted (95%CI [0.31%, 0.33%]). This value of 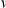 is significantly lower than the average pairwise distance between two members of *enterica* which is around 1% [12]. The same applies to the distribution of genetic diversity introduced by recombination events (Figure S5). This observation goes against the natural tendency of ClonalFrame which is to identify more readily events between distantly related types [40], [41], and therefore indicates that recombination happened predominantly between related strains during the evolution of *enterica*, with recombination between distinct lineages being rarer.

**Figure 4:**
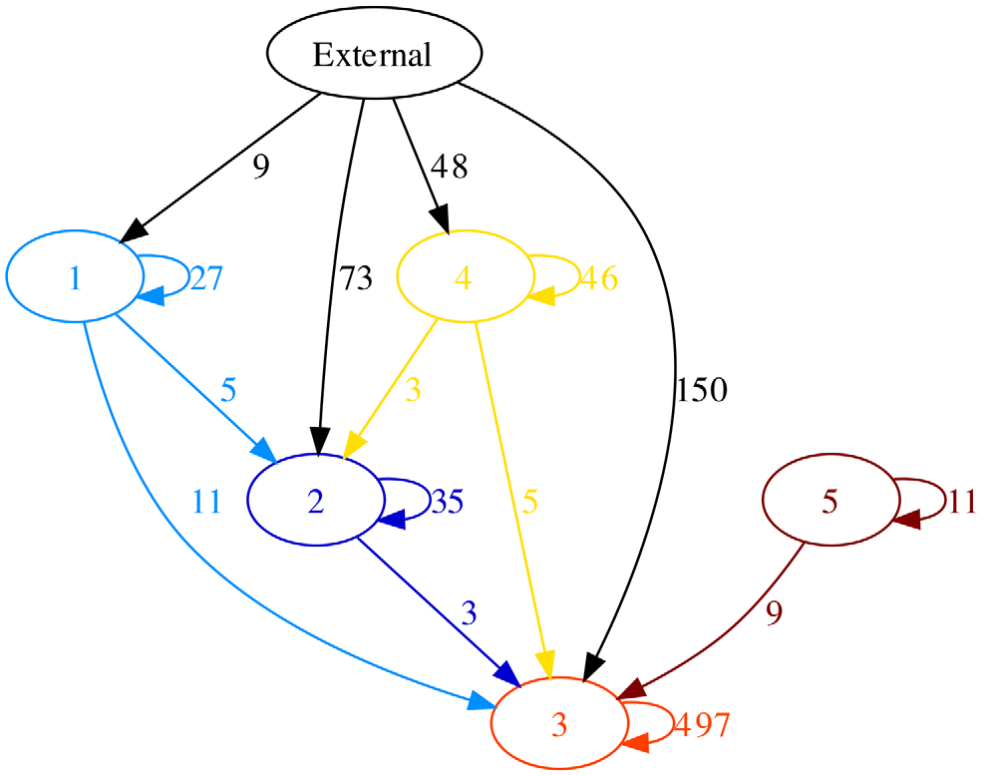
Recombination flux reconstructed between the five lineages. The numbers next to each edge represent the number of recombination events coming from a given origin into a given lineage. Edges with less than 3 events have been omitted. This figure was drawn using GraphViz [84].

We attempted to attribute an origin to each recombination event found by ClonalFrame in the five lineages following the method of [41]. Table S3 shows the events for which an origin could be unambiguously attributed, and Figure 4 illustrates the flux of recombination between the five lineages as well as the events coming from other origins within *enterica*. In lineages 1, 3 and 5, the majority of events was found to come from within these lineages even if ClonalFrame is predisposed to underestimate the propensity of such events [40]. In lineages 2 and 4 however, the primary source of recombination events was “External”, i.e. not contained within one of the five lineages (Figure 4). The origin of these events was not attributed to any isolate or group of isolates in particular, but seemed to come fairly uniformly from all parts of *enterica* minus the five lineages.

## Discussion

### Delineation of *enterica*

We have sequenced approximately one tenth of the core genome from 114 isolates of *enterica* from global sources in order to study its population structure. We identified five clear lineages, defined as groups of isolates having the same majority of ancestry in the Structure analysis and representing a clade in the ClonalFrame analysis. It is likely that other similar lineages exist and would be identified using a larger sample of strains. For example, the four strains of serovar Heidelberg (labelled 44, 45, 70 and 81) were closely related to each other (Figure 3) and would probably have been called a lineage in our analysis if our sample had contained one or two more similar isolates, since lineage 5 was reconstructed based on only 5 isolates (Table 1). Our analysis did not include any isolate of serovar Typhi, which has previously been shown based on whole-genome comparisons to be highly monomorphic [19], [24], [36] and unrelated to other serovars [22], [45]. In the context of the *enterica* data reported here, Typhi would thus constitute a separate and independent lineage, with all current Typhi samples descended from a recent common ancestor on this lineage.

One of the five lineages we identified is particularly ancient, estimated to be two thirds of the age of *enterica*. In the absence of an internal mutation rate for *enterica* [46], it is currently not possible to date this age in terms of years. This ancient lineage was designated as “clade B” in a previous study based on MLST [12], which also noted that it might represent the deepest lineage within *enterica* but that MLST data was insufficient to confirm this hypothesis. Here we provide such data and confirm the existence of this lineage. The identification of this deep lineage is in sharp contrast with a lack of resolution in the deep ancestry of *enterica* in general (Figure 3). A star-shaped phylogeny had also been reconstructed before based on MLST data [12]. Two non-mutually exclusive hypotheses can be proposed to explain this observation: a loss of information about clonal relationships due to extensive recombination [47], and the fast growth of the effective population size shortly following the birth of the population [48].

### Patterns of recombination in *enterica*

It is now clear that recombination plays a driving role in the evolution of many bacteria [15], including *Salmonella* [14]. It has been noted that recombination happens more often within the subspecies of *Salmonella enterica* than between members of separate subspecies [13], but little is known about the details of the recombination process within subspecies *enterica*. A recent study based on MLST data hinted at an unusually high rate of recombination between the Newport-II and Newport-III groups [11]. However, the number of recombination events detectable with MLST is generally too small to draw hard conclusions about rates of recombination. Here we sequenced a hundred times more data per isolate than MLST, which allowed us to reconstruct many recombination events, thus revealing clear patterns. We found evidence for recombination that varied over at least an order of magnitude across lineages of *enterica* (Table 1). Different recombination rates for individual lineages of a same species have been found previously between the seroresistant and serosensitive clades of *Moraxella catarrhalis* [49], between lineages I and II of *Listeria monocytogenes* [50], [51], and between the six hypervirulent lineages of *Neisseria meningitidis* [27]. It is likely that more examples will be found in future studies as improved methods for detecting recombination are applied to large datasets of whole genomes [52].

Recombination events that occurred between distantly related bacteria are easier to detect than events involving close relatives, because they introduce more polymorphism. ClonalFrame is especially biased against the detection of intra-lineage recombination, because it is based on a model of extra-population recombination [40]. In spite of this, we found that recombination was predominantly between members of a lineage in at least three of the five lineages (Figure 4). At least three hypotheses can be formulated to explain this general pattern. Firstly, certain serovars of *enterica* are restricted or associated with specific host species [3] which may result in greater opportunities for recombination between related strains, as previously described in *Campylobacter jejuni* [53]. For instance, lineage 5 consists of isolates of Paratyphi A and Sendai which are restricted to infecting humans [20], [22]. However, lineage 1 contains serovars Choleraesuis, Paratyphi C and Typhisuis which share the same antigenic formula but are differentially adapted to infecting swine, humans and swine, respectively [54]. The other three lineages contain isolates from serovars that are usually described as ubiquitous [3]. Secondly, imports from a distant source might reduce the fitness of the recipients and therefore be removed by selection. Thirdly, laboratory experiments have shown that in many bacteria the chances of success of an import decrease exponentially with the genetic distance between donor and recipient due to the DNA mismatch repair system [55], [56]. This decrease is particularly strong in *enterica*, with recombination between Typhi and Typhimurium reported to be 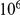 times less likely than within Typhimurium [57], [56]. The predominance of recombination events within lineages could thus reflect a fundamental property of recombination rather than ecological structuring or selection.

### Speciation in *enterica*

The genus *Salmonella* is now generally accepted to contain two species, *S. bongori* and *S. enterica*, the latter of which consists of six subspecies including subspecies *enterica* which is the subject of the present study [58], [59]. Many previously named species that had been defined on the basis of phenotypic differences were regrouped into the single species *S. enterica* on the basis of DNA hybridization results [60].

The difficulty in defining bacterial species stems from our lack of understanding of the processes involved in their formation [61]. Recombination plays a cohesive role in bacteria, so that lineages can evolve into separate species only if recombination is rare between members of distinct lineages [56], [62]. Computer simulations have shown that reduced recombination between lineages can lead to patterns of genetic diversity that are similar to those observed in nature [12], [63]. Our reconstruction of recombination flux within and between the five lineages of *enterica* (Figure 4) strongly supports the existence of barriers to recombination between members of separate lineages. It is therefore possible that the five lineages we identified in *enterica* represent incipient species which have already diverged too far from each other for recombination to regroup them. Such incipient species have the potential to eventually become separate species unless an important shift in genetic flow occurred like the one that was recently reported between *Campylobacter jejuni* and *coli* [64].

Many biological models of bacterial speciation have been proposed in the literature, and it is interesting although speculative to ask ourselves which ones apply to the diversification pattern we described in *enterica*. Under a strict host-association, speciation would be expected to happen through the periodic selection model where adaptation to a host progressively drives between-lineages divergence whilst constraining the genetic diversity of each lineage [65], [66]. This model might apply to lineage 5 which contains serovars restricted to humans, but is unlikely to apply to the other four lineages which can be found in a range of hosts. Alternatively, speciation in *enterica* could be driven by co-evolution with certain bacteriophages which have been shown to infect some serovars more readily than others [67]. Under the geographic mosaic model [68], [69], such uneven adaptive pressures can increase the rate of divergence between populations, and this effect was demonstrated in laboratory experiments on *Pseudomonas fluorescens* [70]. Future research aimed at testing the geographic mosaic theory will need to investigate whether the underlying process is relevant to the evolution of *enterica* [71].

### Comparing Structure and ClonalFrame

The results we have described were obtained using two popular analytical tools: Structure [38] and ClonalFrame [40], which are based on very different evolutionary models. Structure assumes that each individual in the sample is a mixture from a number of unrelated ancestral populations. ClonalFrame assumes that the individuals are related via a phylogenetic framework, but that clonal relationships are occasionally obscured by recombination events. Clearly the Structure model makes more sense for highly recombinogenic species (for example *H. pylori*; [72]) and the ClonalFrame model for mostly clonal bacteria (for example *Yersinia pestis*; [73]). However, for many species including *Salmonella enterica*, recombination occurs but is not sufficiently frequent to completely erase all clonal relationships. Species with such intermediate population structure are eminently suitable for analysis by both models.

We have demonstrated that a combined approach using both methods can aid interpretations of population structure and ancestry. In order to study genetic flux, we needed to first define lineages on the ClonalFrame phylogeny (Figure 3), and Structure allowed us to determine which clades represent meaningful populations. Conversely, the clustering by Structure (Figure 2) could easily have been misinterpreted in the absence of the phylogenetic information provided by ClonalFrame. Structure suggested the existence of a sixth population which seemed to be both a frequent donor and recipient of recombination events (Figure 2). This sixth population is in fact a random mixture of all “other” strains that did not fall into one of the five true lineages (Figure 3) and therefore does not represent a real evolutionary lineage. We therefore interpret this sixth population as an artifact and do not believe that it represents a true evolutionary lineage. In interpreting the levels of mixed ancestry of these five lineages it is also important to note their different relative ages (Figure 3; Table 1). Older lineages will have had more opportunities for recombination than recent ones, resulting in greater admixture in some lineages than in others. Once the outputs of the two methods were interpreted correctly in the light of each other, it became clear that they were in good agreement and allowed a more detailed and trustworthy analysis than each approach would have allowed on its own.

## Materials and Methods

### Bacterial isolates

We analysed a total of 114 previously described isolates of *enterica* including nine from the *Salmonella* reference collection A (SARA; [74]), and 63 of the 72 strains in the *Salmonella* reference collection B (SARB; [75]). The isolates were chosen to span the global diversity of *enterica* as measured by serotyping and MLST. Table S1 contains the full list of the 114 isolates, including their serotype and Sequence Type (ST) in the MLST scheme of [9]. A database of isolates that have been typed using this MLST scheme is accessible at http://mlst.ucc.ie/mlst/dbs/Senterica.

### Choice of genomic regions to sequence

The genome of Typhimurium LT2 [17] was aligned using Mauve [76], [77] against the following ten publicly available genomes from the Genomes OnLine Database (accessible at http://www.genomesonline.org; [25]): Choleraesuis [21], Dublin (University of Illinois, unpublished), Pullorum (University of Illinois, unpublished), Paratyphi A [20], Paratyphi B (University of Washington, unpublished), Typhi CT18 [18], Enteritidis PT4 [78], Gallinarum [78], Hadar (Sanger Institute, unpublished) and Infantis (Sanger Institute, unpublished). The black circle on Figure 1 shows the proportion of these ten genomes that aligned to various parts of the LT2 genome. We selected 146 regions of length 2000-2500bp each from the core genome of *enterica* where at least nine of the ten genomes aligned with LT2. The regions were selected to be distributed evenly around the genome of LT2 (Figure 1), and to include the location of the MLST fragments of the scheme of [9]. This allowed an assessment of the accuracy of the sequencing and direct assessment of analysis based on MLST data. Table S2 contains the location and gene content of each region.

### Resequencing scheme

We designed an Affymetrix CustomSeq Resequencing Array to sequence each of the 114 isolates in Table S1 across the 146 genomic regions listed in Table S2. The reference genome on the microarray was generated by *in silico* optimisation of the probability of accurately resequencing the 11 genomes above. Briefly, we started with the genome of LT2 as reference, proposed iterative changes accepted only when they decreased the chance of having two differences within 25 bp between the reference and one of the 11 genomes (which might make them more difficult to call), and repeated the process until convergence. Tests performed on an earlier version of our resequencing array showed that such an optimised reference performed better than using the genome of LT2 as reference in terms of both calling and error rates (data not shown). Base calling was performed using the Affymetrix GeneChip Sequence Analysis Software (GSEQ). We excluded the GSEQ calls of differences from the reference sequence which were within 13 bp of each other. Such calls are unreliable because hybridization at the central position of a probe can be affected by additional differences in the flanking 12 bp. Our resequenced data is available from http://www.stats.ox.ac.uk/lab/salmonella.zip.

### Structure analysis

We used the Bayesian analysis tool Structure version 2.3 [38] to identify the populations present in our data. The linkage model of Structure was used; this explicitly accounts for the correlation between nearby sites that arise in admixed populations [39]. Four independent runs were performed for each value of the number of populations 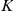 ranging from 2 to 10. Each run consisted of 100,000 MCMC iterations, of which the first half was discarded as burn-in. Convergence and mixing of the program were found to be acceptable by manual comparison of independent runs with the same value of 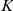. The optimal value was found to be 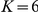 by comparing the posterior probabilities of the data given each value of 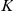 from 2 to 10 (Figure S1), and identifying the value of 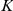 where the posterior probabilities plateau as described in [79]. Applying the method of [80] also resulted in the estimate 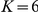 (Figure S2).

### ClonalFrame analysis

We applied the analysis tool ClonalFrame version 1.2 [40] to our data. ClonalFrame is a Bayesian inference method which jointly reconstructs the clonal relationships between the isolates in a sample, as well as the location of recombination events that have disrupted the clonal signal. Four independent runs of ClonalFrame were performed each consisting of 200,000 MCMC iterations, and the first half was discarded as burn-in. Convergence and mixing of the MCMC were found to be satisfactory by manual comparison of the runs and using the method in [81]. The genealogies estimated by ClonalFrame have branch lengths measured in coalescent units of time, which are equal to the effective population size 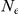 times the duration of a generation. We multiplied this by the posterior means of the scaled mutation rate 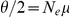 and the scaled recombination rate 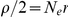 in order to have branch lengths measured in terms of the expected number of mutation and recombination events (where 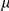 and 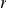 are the per-generation rates of mutation and recombination).

### Attribution of origins to the ClonalFrame recombination events

For each branch of the tree reconstructed by ClonalFrame, we extracted the fragments that had a posterior probability of recombination above 0.5 throughout and which reached 0.95 in at least one position. Each such recombined fragment was then compared with the homologous sequence of all isolates other than those below the affected branch as described [41]. If a match was found with 0 or 1 difference, the origin of the recombination was attributed to the lineage to which the matching isolate belongs. If no match was found, or if several isolates from different lineages matched, the origin of the recombined fragment was considered unresolved.

## Supporting Information

Figure S1 Posterior probability of the number of populations in Structure.
(PDF)

Figure S2 Procedure of Evanno et al. (2005) to determine the number of populations in Structure.
(PDF)

Figure S3 Result of Structure based on MLST data only.
(PDF)

Figure S4 Result of ClonalFrame based on MLST data only.
(PDF)

Figure S5 Distribution of genetic diversity introduced by recombination events in ClonalFrame.
(PDF)

Table S1 List of isolates.
(PDF)

Table S2 List of sequenced regions.
(PDF)

Table S3 Recombination flux between and within lineages.
(PDF)

## References

[1] Kauffmann F (1975). Classification of bacteria: a realistic scheme with special reference to the classification of *Salmonella* and *Escherichia* species..

[2] Grimont P, Weill F (2007). Antigenic formulae of the *Salmonella* serovars, 9th Edition..

[3] Uzzau S, Brown DJ, Wallis T, Rubino S, Leori G (2000). Host adapted serotypes of *Salmonella enterica*.. Epidemiol Infect.

[4] Galanis E, Lo Fo Wong DM, Patrick ME, Binsztein N, Cieslik A (2006). Web- based surveillance and global *Salmonella* distribution, 2000-2002.. Emerg Infect Dis.

[5] Beltran P, Musser JM, Helmuth R, Farmer JJ, Frerichs WM (1988). Toward a population genetic analysis of *Salmonella*: genetic diversity and relationships among strains of serotypes *S. choleraesuis, S. derby, S. dublin, S. enteritidis, S. heidelberg, S. infantis, S. newport, and S. typhimurium*.. Proceedings of the National Academy of Sciences of the United States of America.

[6] Selander RK, Beltran P, Smith NH, Helmuth R, Rubin FA (1990). Evolutionary genetic relationships of clones of *Salmonella* serovars that cause human typhoid and other enteric fevers.. Infect Immun.

[7] Maiden MCJ, Bygraves JA, Feil E, Morelli G, Russell JE (1998). Multilocus sequence typing: A portable approach to the identification of clones within populations of pathogenic microorganisms.. PNAS.

[8] Maiden MC (2006). Multilocus sequence typing of bacteria.. Annual Review of Microbiology.

[9] Kidgell C, Reichard U, Wain J, Linz B, Torpdahl M (2002). *Salmonella typhi*, the causative agent of typhoid fever, is approximately 50,000 years old.. Infect Genet Evol.

[10] Torpdahl M, Skov MN, Sandvang D, Baggesen DL (2005). Genotypic characterization of *Salmonella* by multilocus sequence typing, pulsed-field gel electrophoresis and amplified fragment length polymorphism.. J Microbiol Methods.

[11] Sangal V, Harbottle H, Mazzoni CJ, Helmuth R, Guerra B (2010). Evolution and population structure of *Salmonella* enterica serovar Newport.. J Bacteriol.

[12] Falush D, Torpdahl M, Didelot X, Conrad DF, Wilson DJ (2006). Mismatch induced speciation in *Salmonella*: model and data.. Phil Trans R Soc B.

[13] Brown EW, Mammel MK, LeClerc JE, Cebula TA (2003). Limited boundaries for extensive horizontal gene transfer among *Salmonella* pathogens.. Proc Natl Acad Sci.

[14] Octavia S, Lan R (2006). Frequent recombination and low level of clonality within *Salmonella* enterica subspecies I.. Microbiology.

[15] Didelot X, Maiden MC (2010). Impact of recombination on bacterial evolution.. Trends Microbiol.

[16] Tenaillon O, Skurnik D, Picard B, Denamur E (2010). The population genetics of commensal *Escherichia coli*.. Nature Reviews Microbiology.

[17] McClelland M, Sanderson KE, Spieth J, Clifton SW, Latreille P (2001). Complete genome sequence of *Salmonella enterica* serovar Typhimurium LT2.. Nature.

[18] Parkhill J, Dougan G, James KD, Thomson NR, Pickard D (2001). Complete genome sequence of a multiple drug resistant *Salmonella enterica* serovar Typhi CT18.. Nature.

[19] Deng W, Liou SR, Plunkett G, Mayhew GF, Rose DJ (2003). Comparative genomics of *Salmonella enterica* serovar Typhi strains Ty2 and CT18.. J Bacteriol.

[20] McClelland M, Sanderson KE, Clifton SW, Latreille P, Porwollik S (2004). Comparison of genome degradation in Paratyphi A and Typhi, human-restricted serovars of *Salmonella enterica* that cause typhoid.. Nat Genet.

[21] Chiu CH, Tang P, Chu C, Hu S, Bao Q (2005). The genome sequence of *Salmonella enterica* serovar Choleraesuis, a highly invasive and resistant zoonotic pathogen.. Nucleic Acids Res.

[22] Didelot X, Achtman M, Parkhill J, Thomson NR, Falush D (2007). A bimodal pattern of relatedness between the *Salmonella* Paratyphi A and Typhi genomes: Convergence or divergence by homologous recombination?. Genome Res.

[23] Roumagnac P, Weill FX, Dolecek C, Baker S, Brisse S (2006). Evolutionary History of *Salmonella* Typhi.. Science.

[24] Holt KE, Parkhill J, Mazzoni CJ, Roumagnac P, Weill FX (2008). High-throughput sequencing provides insights into genome variation and evolution in *Salmonella* Typhi.. Nat Genet.

[25] Liolios K, Chen IMA, Mavromatis K, Tavernarakis N, Hugenholtz P (2010). The Genomes On Line Database (GOLD) in 2009: status of genomic and metagenomic projects and their associated metadata.. Nucleic Acids Research.

[26] Falush D (2009). Toward the Use of Genomics to Study Microevolutionary Change in Bacteria.. PLoS Genet.

[27] Didelot X, Urwin R, Maiden MCJ, Falush D (2009). Genealogical typing of Neisseria *meningitidis*.. Microbiology.

[28] Bains W, Smith GC (1988). A novel method for nucleic acid sequence determination.. J Theor Biol.

[29] Zwick ME, Mcafee F, Cutler DJ, Read TD, Ravel J (2005). Microarray-based resequencing of multiple *Bacillus anthracis* isolates.. Genome Biol.

[30] Sougakoff W, Rodrigue M, Truffot-Pernot C, Renard M, Durin N (2004). Use of a high-density DNA probe array for detecting mutations involved in rifampicin resistance in *Mycobacterium tuberculosis*.. Clin Microbiol Infect.

[31] Zwick ME, Kiley MP, Stewart AC, Mateczun A, Read TD (2008). Genotyping of *Bacillus cereus* Strains by Microarray-Based Resequencing.. PLoS ONE.

[32] Dunman PM, Mounts W, McAleese F, Immermann F, Macapagal D (2004). Uses of *Staphylococcus aureus* GeneChips in genotyping and genetic composition analysisic composition analysis.. J Clin Microbiol.

[33] Corless CE, Kaczmarski E, Borrow R, Guiver M (2008). Molecular characterization of *Neisseria meningitidis* isolates using a resequencing DNA microarray.. J Mol Diagn.

[34] Pandya GA, Holmes MH, Petersen JM, Pradhan S, Karamycheva SA (2009). Whole genome single nucleotide polymorphism based phylogeny of *Francisella tularensis* and its application to the development of a strain typing assay.. BMC Microbiol.

[35] Octavia S, Lan R (2007). Single-nucleotide-polymorphism typing and genetic relationships of *Salmonella enterica* serovar Typhi isolates.. J Clin Microbiol.

[36] Achtman M (2008). Evolution, population structure, and phylogeography of genetically monomorphic bacterial pathogens.. Annu Rev Microbiol.

[37] Octavia S, Lan R (2009). Multiple-locus variable-number tandem-repeat analysis of *Salmonella enterica* serovar Typhi.. J Clin Microbiol.

[38] Pritchard J, Stephens M, Donnelly PJ (2000). Inference of population structure using multilocus genotype data.. Genetics.

[39] Falush D, Stephens M, Pritchard J (2003). Inference of population structure using multilocus genotype data linked loci and correlated allele frequencies.. Genetics.

[40] Didelot X, Falush D (2007). Inference of Bacterial Microevolution Using Multilocus Sequence Data.. Genetics.

[41] Didelot X, Barker M, Falush D, Priest F (2009). Evolution of pathogenicity in the *Bacillus cereus* group.. Systematic and Applied Microbiology.

[42] Anjum MF, Marooney C, Fookes M, Baker S, Dougan G (2005). Identification of Core and Variable Components of the *Salmonella enterica* Subspecies I Genome by Microarray.. Infect Immun.

[43] Milkman R, Bridges MM (1990). Molecular Evolution of the Escherichia coli Chromosome. III. Clonal Frames.. Genetics.

[44] Feil E, Maiden M, Achtman M, Spratt B (1999). The relative contributions of recombination and mutation to the divergence of clones of *Neisseria meningitidis*.. Mol Biol Evol.

[45] Holt KE, Thomson NR, Wain J, Langridge GC, Hasan R (2009). BMC Genomics.. BMC Genomics.

[46] Morelli G, Didelot X, Kusecek B, Schwarz S, Bahlawane C (2010). Microevolution of *Helicobacter pylori* during prolonged infection of single hosts and within families.. PLoS Genet.

[47] Schierup MH, Hein J (2000). Consequences of recombination on traditional phylogenetic analysis.. Genetics.

[48] Fiala KL, Sokal RR (1985). Factors determining the accuracy of cladogram estimation evaluation using computer-simulation.. Evolution.

[49] Wirth T, Morelli G, Kusecek B, van Belkum A, van der Schee C (2007). The rise and spread of a new pathogen: seroresistant *Moraxella catarrhalis*.. Genome Res.

[50] den Bakker H, Didelot X, Fortes E, Nightingale K, Wiedmann M (2008). Lineage specific recombination rates and microevolution in *Listeria monocytogenes*.. BMC Evolutionary Biology.

[51] Orsi R, Sun Q, Wiedmann M (2008). Genome-wide analyses reveal lineage specific contributions of positive selection and recombination to the evolution of *Listeria monocytogenes*.. BMC Evolutionary Biology.

[52] Didelot X, Lawson D, Darling A, Falush D (2010). Inference of homologous recombination in bacteria using whole-genome sequences.. Genetics.

[53] McCarthy N, Colles F, Dingle K, Bagnall M, Manning G (2007). Population genetic approaches to assigning the source of human pathogens: host associated genetic import in *Campylobacter jejuni*.. Emerging infectious diseases.

[54] Liu WQ, Feng Y, Wang Y, Zou QH, Chen F (2009). Salmonella paratyphi C: genetic divergence from Salmonella choleraesuis and pathogenic convergence with *Salmonella typhi*.. PLoS ONE.

[55] Majewski J (2001). Sexual isolation in bacteria.. FEMS microbiology letters.

[56] Fraser C, Hanage W, Spratt B (2007). Recombination and the nature of bacterial speciation.. Science.

[57] Zahrt TC, Maloy S (1997). Barriers to recombination between closely related bacteria: MutS and RecBCD inhibit recombination between *Salmonella typhimurium* and *Salmonella typhi*.. Proc Natl Acad Sci U S A.

[58] Tindall BJ, Grimont PA, Garrity GM, Euzéby JP (2005). Nomenclature and taxonomy of the genus *Salmonella*.. Int J Syst Evol Microbiol.

[59] Heyndrickx M, Pasmans F, Ducatelle R, Decostere A, Haesebrouck F (2005). Recent changes in *Salmonella* nomenclature: the need for clarification.. Vet J.

[60] Crosa J, Brenner D, Ewing W, Falkow S (1973). Molecular relationships among the Salmonellae.. J Bacteriol.

[61] Achtman M, Wagner M (2008). Microbial diversity and the genetic nature of microbial species.. Nature Reviews Microbiology.

[62] Fraser C, Alm EJ, Polz MF, Spratt BG, Hanage WP (2009). The bacterial species challenge: making sense of genetic and ecological diversity.. Science.

[63] Hanage WP, Spratt BG, Turner KME, Fraser C (2006). Modelling bacterial speciation.. Phil Trans R Soc B.

[64] Sheppard S, McCarthy N, Falush D, Maiden M (2008). Convergence of *Campylobacter* Species: Implications for Bacterial Evolution.. Science.

[65] Cohan FM (2001). Bacterial species and speciation.. Systematic biology.

[66] Cohan FM, Perry EB (2007). A systematics for discovering the fundamental units of bacterial diversity.. Curr Biol.

[67] Carey-Smith GV, Billington C, Cornelius AJ, Hudson JA, Heinemann JA (2006). Isolation and characterization of bacteriophages infecting salmonella spp.. FEMS Microbiol Lett.

[68] Thompson J (1999). Specific hypotheses on the geographic mosaic of coevolution..

[69] Thompson J (2005). The geographic mosaic of coevolution..

[70] Buckling A, Rainey PB (2002). The role of parasites in sympatric and allopatric host diversification.. Nature.

[71] Gomulkiewicz R, Drown DM, Dybdahl MF, Godsoe W, Nuismer SL (2007). Dos and don'ts of testing the geographic mosaic theory of coevolution.. Heredity.

[72] Falush D, Wirth T, Linz B, Pritchard JK, Stephens M (2003). Traces of human migrations in *Helicobacter pylori* populations.. Science.

[73] Morelli G, Song Y, Mazzoni CJ, Eppinger M, Roumagnac P (2010). *Yersinia pestis* genome sequencing identifies patterns of global phylogenetic diversity.. Nat Genet.

[74] Beltran P, Plock SA, Smith NH, Whittam TS, Old DC (1991). Reference collection of strains of the *Salmonella typhimurium* complex from natural populations.. J Gen Microbiol.

[75] Boyd EF, Wang FS, Beltran P, Plock SA, Nelson K (1993). *Salmonella reference* collection B (SARB): strains of 37 serovars of subspecies I.. J Gen Microbiol.

[76] Darling AC, Mau B, Blattner FR, Perna NT (2004). Mauve: multiple alignment of conserved genomic sequence with rearrangements.. Genome Res.

[77] Darling A, Mau B, Perna N (2010). progressiveMauve: Multiple Genome Alignment with Gene Gain, Loss and Rearrangement.. PLoS ONE.

[78] Thomson NR, Clayton DJ, Windhorst D, Vernikos G, Davidson S (2008). Comparative genome analysis of *Salmonella* Enteritidis PT4 and *Salmonella* Gallinarum 287/91 provides insights into evolutionary and host adaptation pathways.. Genome Res.

[79] Pritchard JK, Wena X, Falush D (2009). Documentation for structure software: Version 2.3,. http://pritch.bsd.uchicago.edu/structure.html.

[80] Evanno G, Regnaut S, Goudet J (2005). Detecting the number of clusters of individuals using the software structure: a simulation study.. Mol Ecol.

[81] Gelman A, Rubin DB (1992). Inference from iterative simulation using multiple sequences.. Statistical Science.

[82] Carver T, Thomson N, Bleasby A, Berriman M, Parkhill J (2009). DNAPlotter: circular and linear interactive genome visualization.. Bioinformatics.

[83] Rambaut A (2008). FigTree, a graphical viewer of phylogenetic trees,. http://tree.bio.ed.ac.uk/software/figtree/.

[84] Gansner ER, North SC (2000). An open graph visualization system and its applications to software engineering.. Software — Practice and Experience.

