# Supplementary figures and images for: Recombination and Population Structure in Salmonella enterica
Source: PLoS Genet. 2011 Jul 28;7(7):e1002191. doi: 10.1371/journal.pgen.1002191 (PMC3145606; doi:10.1371/journal.pgen.1002191)

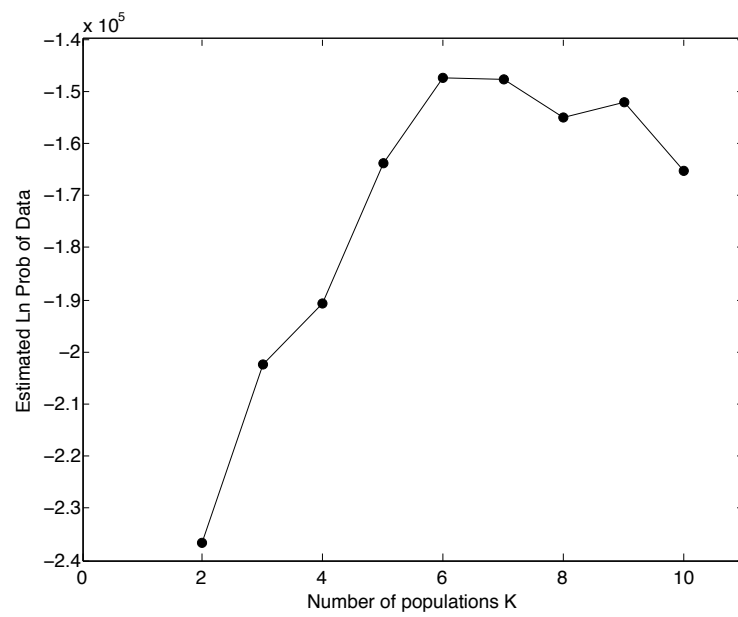

Supplement: Figure S1 — Posterior probability of the number of populations in Structure. (PDF) [file pgen.1002191.s001.pdf]

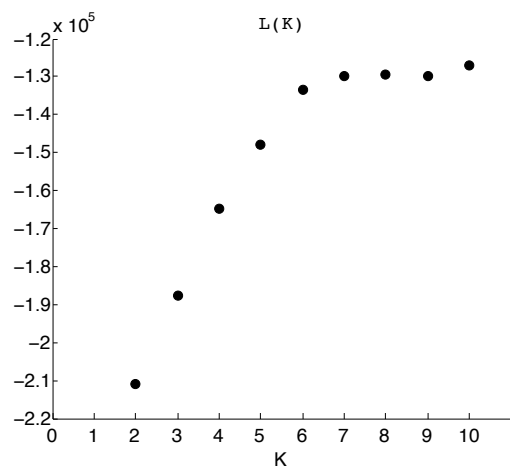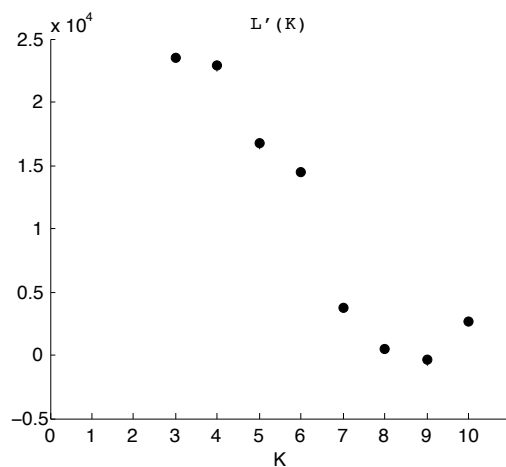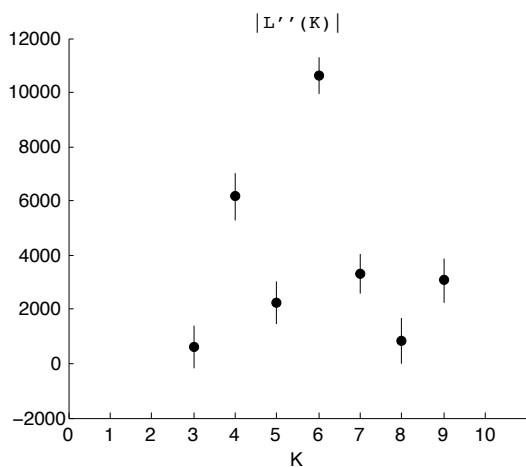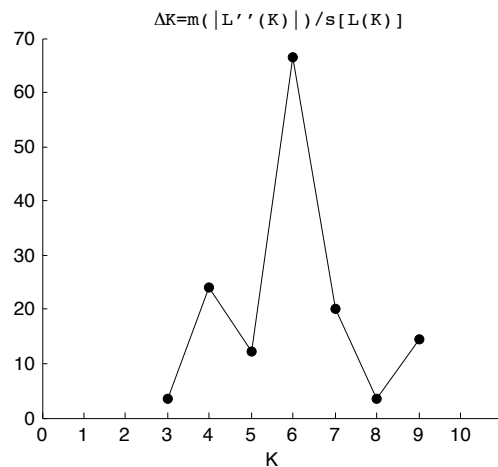

Supplement: Figure S2 — Procedure of Evanno et al. (2005) to determine the number of populations in Structure. (PDF) [file pgen.1002191.s002.pdf]

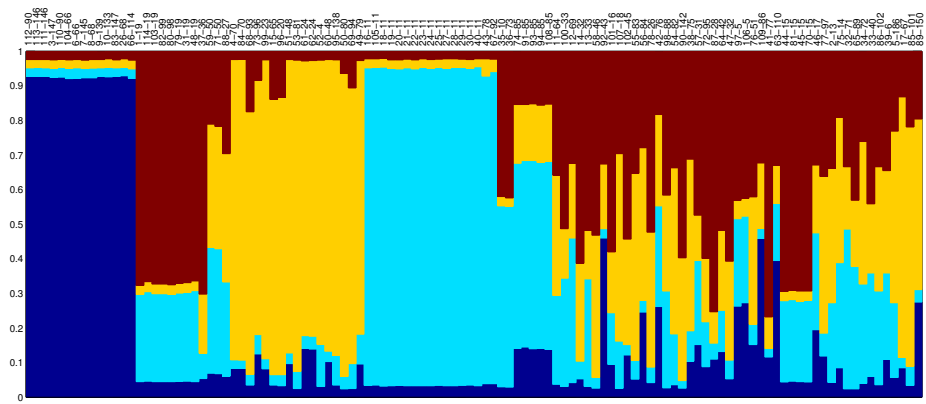

Supplement: Figure S3 — Result of Structure based on MLST data only. (PDF) [file pgen.1002191.s003.pdf]

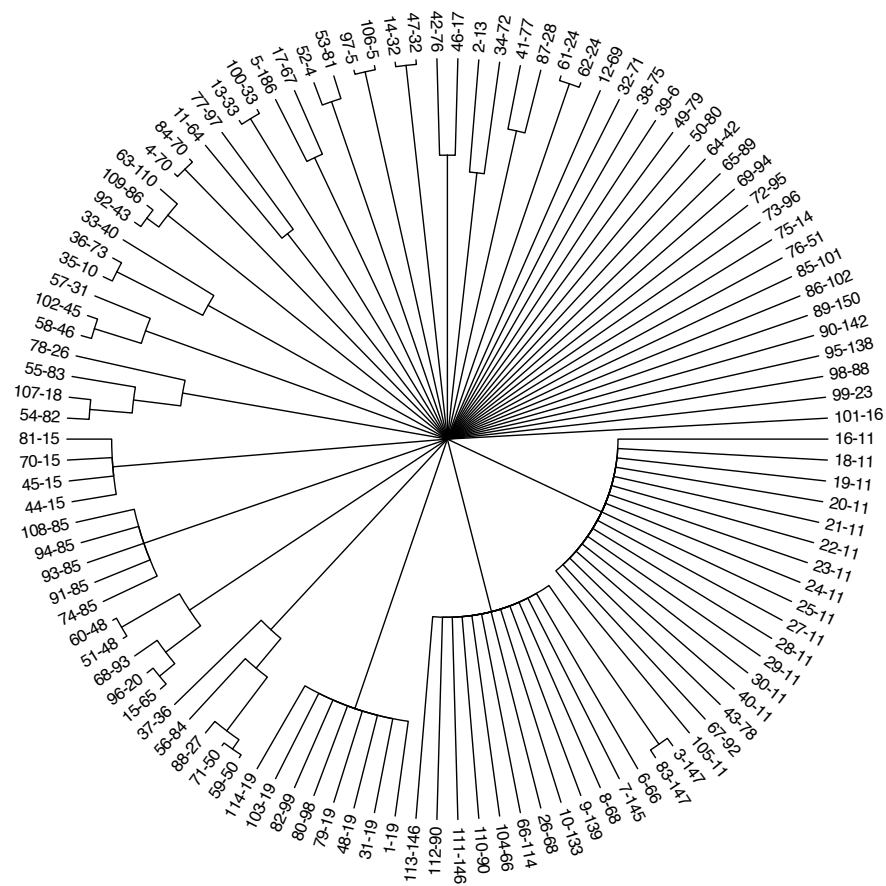

Supplement: Figure S4 — Result of ClonalFrame based on MLST data only. (PDF) [file pgen.1002191.s004.pdf]

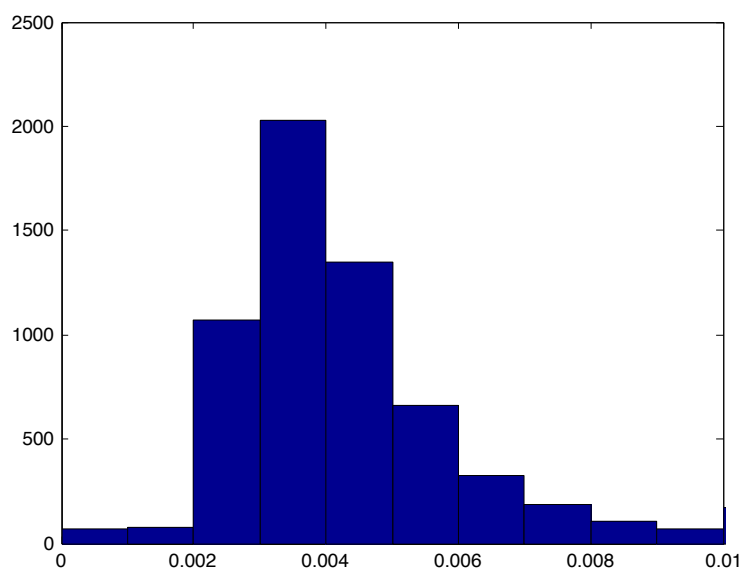

Supplement: Figure S5 — Distribution of genetic diversity introduced by recombination events in ClonalFrame. (PDF) [file pgen.1002191.s005.pdf]
