# Supplementary material for: Recombination and Population Structure in Salmonella enterica
Source: PLoS Genet. 2011 Jul 28;7(7):e1002191. doi: 10.1371/journal.pgen.1002191 (PMC3145606; doi:10.1371/journal.pgen.1002191)
Supplement: Table S1 — List of isolates. (PDF) [file pgen.1002191.s006.pdf]

#	Serovar	Strain	ST
1	Typhimurium	SARA6	19
2	Agona	SARB1	13
3	Typhisuis	61-6	147
4	Decatur	65-3	70
5	Decatur	98-62313	186
6	Choleraesuis	Scb5	66
7	Choleraesuis	RKS1227	145
8	Choleraesuis	RKS1248	68
9	Choleraesuis	49-0554	139
10	Choleraesuis	48-0391	133
11	Anatum	SARB2	64
12	Choleraesuis	SARB7	69
13	Hadar	mto0180	33
14	Infantis	S1326-28	32
15	Brandenburg	SARB3	65
16	Enteritidis	P125109	11
17	Decatur	SARB5	67
18	Enteritidis	VLA_1	11
19	Enteritidis	VLA_2	11
20	Enteritidis	VLA_4	11
21	Enteritidis	VLA_5	11
22	Enteritidis	VLA_7	11
23	Enteritidis	VLA_8	11
24	Enteritidis	VLA_10	11
25	Enteritidis	VLA_12	11
26	Choleraesuis	SARB6	68
27	Enteritidis	VLA_42	11
28	Enteritidis	VLA_45	11
29	Enteritidis	VLA_48	11
30	Enteritidis	VLA_51	11
31	Typhimurium	VLA_21	19
32	Derby	SARB9	71
33	Derby	SARB10	40
34	Derby	SARB11	72
35	Dublin	SARB12	10
36	Dublin	SARB13	73
37	Typhimurium	SARA8	36
38	Duisberg	SARB15	75
39	Enteritidis	SARB17	6
40	Enteritidis	SARB18	11
41	Enteritidis	SARB19	77
42	Emek	SARB20	76
43	Gallinarum	SARB21	78
44	Heidelberg	SARB23	15
45	Heidelberg	SARB24	15
46	Indiana	SARB25	17
47	Infantis	SARB26	32
48	Typhimurium	SARA18	19
49	Infantis	SARB27	79
50	Miami	SARB28	80
51	Miami	SARB29	48
52	Montevideo	SARB30	4
53	Montevideo	SARB31	81
54	Meunchen	SARB32	82
55	Meunchen	SARB33	83
56	Meunchen	SARB34	84
57	Newport	SARB37	31

#	Serovar	Strain	ST
58	Newport	SARB38	46
59	Saintpaul	SARA22	50
60	Panama	SARB39	48
61	Javiana	SARB40	24
62	Javiana	SARB41	24
63	Paratyphi B	SARB44	110
64	Paratyphi B	SARB46	42
65	Limete	SARB47	89
66	Paratyphi C	SARB49	114
67	Gallinarum	SARB51	92
68	Reading	SARB53	93
69	Rubislaw	SARB54	94
70	Heidelberg	SARA35	15
71	Saintpaul	SARB55	50
72	Saintpaul	SARB56	95
73	Schwarzengrund	SARB57	96
74	Sendai	SARB58	85
75	Senftenberg	SARB59	14
76	Stanley	SARB60	51
77	Stanleyville	SARB61	97
78	Thompson	SARB62	26
79	Typhimurium	SARB65	19
80	Typhimurium	SARB66	98
81	Heidelberg	SARA40	15
82	Typhimurium	SARB68	99
83	Typhisuis	SARB69	147
84	Decatur	SARB70	70
85	Wien	SARB71	101
86	Wien	SARB72	102
87	Paratyphi B	00-03009	28
88	Saintpaul	04-00168	27
89	Bovismorbificans	04-00762	150
90	Bovismorbificans	05-00504	142
91	Paratyphi A	MZ763	85
92	Paratyphi B	SARA47	43
93	Paratyphi A	MZ726	85
94	Paratyphi A	MZ755	85
95	Montevideo	MZ773	138
96	Saintpaul	MZ774	20
97	Newport	MZ784	5
98	Paratyphi B	SARA56	88
99	Oranienburg	00-02036	23
100	Hadar	mto174	33
101	Virchow	00-02323	16
102	Newport	RH01-3000	45
103	Typhimurium	SARA11	19
104	Choleraesuis	SARB4	66
105	Enteritidis	SARB16	11
106	Newport	SARB36	5
107	Manhattan	SARB35	18
108	Paratyphi A	SARB42	85
109	Paratyphi B	SARB43	86
110	Paratyphi C	SARB48	90
111	Paratyphi C	E44	146
112	Paratyphi C	E700	90
113	Paratyphi C	E702	146
114	Typhimurium	NCTC13348	19
