# Supplementary material for: Recombination and Population Structure in Salmonella enterica
Source: PLoS Genet. 2011 Jul 28;7(7):e1002191. doi: 10.1371/journal.pgen.1002191 (PMC3145606; doi:10.1371/journal.pgen.1002191)
Supplement: Table S2 — List of sequenced regions. (PDF) [file pgen.1002191.s007.pdf]

Start	End	Gene content
137	2437	thrL thrA
10337	12336	htgA yaaI dnaK
17344	19343	
22355	24354	
40183	42182	
46912	48911	nhaA nhaR
61541	63540	
81930	83929	caiD caiC
84995	86994	caiB caiA caiT
132791	134790	leuA leuL leuO
165853	167852	hofC hofB ppdD
247079	249078	cdaR yaeH
295041	297040	yafB yafC
300709	302708	dniR gloB yafS
345848	347847	safC safD ybeJ
352217	354216	yafV fadE
364662	366661	crl phoE proB
413258	415257	foxA yahN
427354	429353	yaiU yaiV ampH
432888	434887	yaiY yaiZ ddl
457583	459582	malZ yajB queA
511385	513384	ybaW ybaX ybaE
524321	526320	ybaZ ffs ylaB
531175	533174	acrB acrA
575185	577184	ybbS allA allR
596175	598574	purK purE lpxH ppiB
640449	642448	ybdK entD fepA
667192	669191	ybdN ybdO
676301	678300	ybdQ ybdR
801345	803845	sucA
809101	811100	cydA
865967	867966	uvrB slrP
895921	897920	glnP glnH dps
954600	956599	ulaA
1048822	1050821	dmsC ycaD
1055782	1057781	pflB focA
1176957	1178956	pipB pipC sopB
1195656	1197655	hpaA
1221501	1223500	
1230749	1232748	csgB csgA csgC ymdA
1234541	1236540	ymdC mdoC mdoG
1241712	1243711	yceE htrB
1312005	1314004	potB potA pepT
1318150	1320149	phoQ phoP purB
1412380	1414379	thrS infC
1417791	1419790	pheT himA btuC
1422470	1424469	nlpC ydiV ydiU
1425599	1427598	aroH ydiA
1451656	1453655	sufA sufB

Start	End	Gene content
1478686	1480685	ssrA ssaB ssaC
1489210	1491209	sseG ssaG ssaH ssaI ssaJ
1493589	1495588	ssaM ssaV
1498280	1500279	ssaQ ssaR ssaS ssaT
1540460	1542459	ydgA manA fumA
1589097	1591096	ydfG dcp
1605332	1607331	
1609024	1611023	
1700105	1702104	ydcK rimL
1731017	1733016	ydcF hrpA
1740013	1742012	ldhA hslJ
1786591	1788590	sapC sapD sapF
1827475	1829474	ompW yciC
1867903	1869902	ychP ychN chaB chaA
1877387	1879386	ipk prsA ychM
1904307	1906306	ycgB fadR
1959316	1961315	pagO
2043536	2045535	fliY fliZ fliA
2050019	2052018	fliD fliS fliT amyA
2071083	2073082	yedI yedA
2101115	2103114	cbiP cbiO cbiQ cbiN cbiM
2115378	2117377	pduF pduA pudB
2130200	2132199	pduT pduU pduV pduW
2150067	2152567	hisG hisD hisC
2157702	2159701	wzzB udg gnd
2228331	2230330	yegQ
2253269	2255268	yehS yehT yehU
2313083	2315082	yeiU spr rtn
2384487	2386486	nrdB yfaE
2429170	2431169	nuoL nuoK nuoJ nuoI nuoH
2471006	2473005	
2474832	2476831	purF cvpA dedD
2493826	2496326	mepA aroC yfcB
2508737	2510736	pgtB pgtC
2514256	2516255	ddg yfdZ
2591106	2593105	aegA narQ
2597041	2599040	yffB dapE
2685036	2687035	suhB asrA
2720263	2722262	yfhL acpS
2723971	2725970	era rncS lepB
3023245	3025244	sptP sicP iacP sipA
3031930	3033929	spaS spaR spaQ spaP
3037721	3039720	invC invB invA
3065445	3067444	rpoS nlpD
3099166	3101165	pyrG mazG
3125369	3127368	fucO fucA
3151004	3153003	ptr recC
3156849	3158848	ppdB ppdA thyA lgt
3209456	3211455	bglA

Start	End	Gene content
3250407	3252406	speA yqgB yqgD metK
3269839	3271838	yggX mltC nupG
3286363	3288362	
3311224	3313223	hybB hybA hypO
3342113	3344112	ygiY mdaB ygiN parE
3352863	3354862	ygiD ygiE
3355922	3357921	ribB
3392444	3394443	ygjT ygjU
3524601	3526600	
3532672	3534671	yhcQ yhcR yhcS tldD
3536125	3538124	yhdP
3563402	3565401	acrF
3586235	3588234	secY rplO rpmD rpsE
3716353	3718352	
3760811	3762810	uspA yhiP
3815323	3817322	dppA
3891901	3893900	
3908665	3910664	rfaL rfaK
3912551	3914550	rfaJ rfaI rfaB
3935470	3937469	spoT spoU recG
3953400	3955399	rmbA misL
3956696	3958695	misL fidL marT
3961549	3963548	mgtB
4041969	4044469	recF dnaN dnaA
4071682	4073681	glmU atpC
4075548	4077547	atpG atpA
4143480	4145911	hemX hemD hemC
4160460	4162459	rarD yigI pldA
4177192	4179191	yigN ubiE yigP aarF
4215736	4217735	glnA typA
4258771	4260770	rhaB rhaS rhaR
4289252	4291251	yneC tpiA yiiQ
4295549	4297548	glpK glpF yiiU
4369648	4371647	rpoB rpoC
4414414	4416413	pepE yjbC yjbD
4444831	4446830	yjbH yjbA malG
4451729	4453728	malK lamB malM
4484020	4486019	
4491551	4493550	
4494917	4496916	
4498570	4500569	
4527581	4529580	lpxO phnO
4534547	4536546	yjdB yjdE
4540711	4542710	adi melR melA
4576347	4578346	groEL yjeI yjeJ
4652378	4654377	
4704993	4706992	pyrL
4804831	4806830	yjjP yjjQ bglJ
