# Supplementary material for: Recombination and Population Structure in Salmonella enterica
Source: PLoS Genet. 2011 Jul 28;7(7):e1002191. doi: 10.1371/journal.pgen.1002191 (PMC3145606; doi:10.1371/journal.pgen.1002191)
Supplement: Table S3 — Recombination flux between and within lineages. (PDF) [file pgen.1002191.s008.pdf]

	Lineage 1	Lineage 2	Lineage 3	Lineage 4	Lineage 5	Others
Lineage 1	27	0	0	1	0	9
Lineage 2	5	35	2	3	1	73
Lineage 3	11	3	497	5	9	150
Lineage 4	2	2	0	46	1	48
Lineage 5	1	0	0	0	11	2
